# Notes and News

**Published:** 1905-08-26

**Authors:** 


### Notes and News.

The epidemic of yellow fever in New Orleans shows no abatement.

The late Mr. John Rotherham has bequeathed £1,000 to the Coventry and Warwickshire Hospital.

A fancy fair and fête, recently held at Bridgewater, has resulted in a contribution of £1,000 to the funds of the infirmary.

The new out-patient department at the Bradford Children's Hospital has been opened recently. It comprises a large waiting hall capable of holding 200 persons, operating-rooms, offices, and curator's residence.

A small-pox hospital has been opened for Grangemouth at Avonside, near Polmont, N.B. It is of composite iron and wood, consists of two wards for four beds, nurses' rooms, kitchen and sanitary block.

The Royal Sanitary Institute will hold a special course of practical training in food and meat inspection for commissioned officers and professional men preparing for the examinations of inspectors of meat and other foods. The course will begin on November 22nd, when Mr. James King, M.R.C.V.S., will commence a course of six lectures and demonstrations. These lectures will be followed by others, to be given by competent authorities.

Mrs. Ellinor Close has not been daunted by the opposition which met her proposal of a scheme to send children under the Poor-law to Canada, to be brought up there, and possibly returned to Great Britain later on as healthy citizens, capable of maintaining themselves. Through her advocacy a City banker has provided the means to purchase a farm in Canada, where the scheme can be tested. Mrs. Close hopes to leave England shortly with a party of children, who will be established in the farm in New Brunswick, and brought up as agriculturists. They will receive free education provided by the Government, and be supervised by a local committee. Mrs. Close hopes to show that such a farmhouse can be made self-supporting.

A new workhouse infirmary is proposed for Plymouth, and it has been decided to extend the Lady Eden Cottage Hospital at Bishop Auckland.

An appeal is being made, especially to those who have visited or are visiting Ober-Ammergau, to provide funds to pay for the services of a nurse for the poor in their own homes in connection with the Ober-Ammergau Cottage Hospital. Miss Milner Heworth, Moor House, York, will supply information or receive contributions.

The Special Appeal Committee of the Royal Waterloo Hospital for Children and Women are asking for funds on behalf of the institution, which has been rebuilding for some time past, and will have cost about £50,000 when finished, of which only £20,000 has been obtained. The offices of the Appeal Committee are at 156 Regent Street, where all contributions may be sent.

The proposal to acquire Gowbarrow Fell and Aira Force, Ulleswater, for the nation is one that must interest all those interested in the national welfare. The population of our large northern towns largely resort to the Lakeland during holiday seasons, and as the size of these towns continues to increase it is essential to maintain as large an area of playground as possible. This reason is sufficient to recommend that the nation should prevent so beautiful a region falling into the hands of the builder.

A "Silly Season" jest has been occupying attention in a portion of the daily press, and excitement has consequently been aroused concerning a reported pirate vessel which was said to have been seen at different places between Trieste and the Solent. The mystery was solved at Ryde, where the sailing boat arrived, and turned out to have been chartered for advertisement purposes by Edward Cook and Sons, the manufacturers of medicinal and other soaps.

The Sanitary Inspectors' Autumn Conference, held at Carpenters Hall, has been largely attended, the president of the Association, Sir J. Crichton Browne, presiding. Dr. W. Daley, who read a paper on Sanitation and Management of Dairies and Cowsheds, expressed his opinion that it was safer to sterilise all milk used as the sole food of children. Mr. J. Brand, Mr. F. Smith, Mr. W. Martin and the President, uttered some very practical suggestions for the proper teaching of the laws of hygiene and sanitation to the public.

The York Corporation Health Committee, acting under the advice of their Medical Officer of Health, Dr. E. M. Smith, are asking the Education Committee to do their utmost to prevent the attendance of children under five years of age at the elementary schools in the city. The advice has been given on account of the infectious diseases which have been spread through the infant schools. The step will not be popular amongst the mothers of these children, who in some cases will be unable to undertake any work if the schools do not look after the younger children.

